# Hospital Reports

**Published:** 1877-01-20

**Authors:** 

**Affiliations:** Resident Physician


### HOSPITAL REPORTS. — PHILADELPHIA DISPENSARY.

DR. MARIS, RESIDENT PHYSICIAN.

REVIEW OF THE TREATMENT OF NERVOUS DISEASES, AND EXTRACTS FROM CLINICS.

NO. I.

REPORTED BY C. C. VANDERBECK, M. D.

### EPILEPSY.

Dr. Maris depends chiefly upon the bromides, large doses and long continued. He has seen but one case that seemed to be actually cured. In the majority of cases there is an improvement, a check of the disease, but his experience leads him to believe that real cure is seldom.

### NEURALGIA.

When there is any history or periodicity, he uses *sulphate of cinchonia.* He began to use this on account of its cheapness compared with quinia, as this is an important point to remember in dispensary service. He is satisfied now, from the evidence of a long experience, and abundant trials, and careful watching of results, that the cinchonia sulphate is *just as efficacious* as quinine, and need not be given in one-third larger doses. It is a drug to be preferred to quinia, as it has not the same tendency to bring on various unpleasant cerebral symptoms. In many cases of neuralgia he is fond of using the bromides. A very favorite prescription, when there is need of any decided narcotic effect, is what is known in the institution as the *narcotic tonic.* The formula of this is as follows:

R. Ext. opii ........................
Ext. conii, ........................
Ext. can. indicæ ...... ........
Ext. bellad ..............aa dr. ss.
Dis. in alcohol ............oz. iss
Add
Strychnia, ...................gr. ss
(dis. in dr. j of alcohol)
Quin. sul.. ..................gr. x
Aq. acidulata ...............oz. j
Ol. anisi. .......................gtt. iij
Sy. acacia ....................oz. j M.

Sig.—Dose 10 drops, repeated cautiously.

Again, the subcarbonate of iron is of peculiar value in very many cases of neuralgia, not simply as a ferruginous tonic in anæmic cases, but it seems to have a special or peculiar effect in this painful malady. He does not attempt to explain the modus operandi, but a multiplied experience has taught him its value, and has corroborated the statement made to him years ago, by old Dr. Casper Wistar, of this city, who claimed the wonderful value of this drug in neuralgia, especially that variety affecting the fifth pair of nerves.

### SCIATICA.

Only a general idea can be given of the plan of treatment for these various nervous diseases. The treatment varies, of course, but this review is to present more particularly any special features of therapeutics. Besides the usual plan of counter-irritation, the doctor is in the habit of prescribing a mixture of cimicifuga and phosphate of ammonia. The prescription is this:

R. Tinct. cimicifugæ.......
Liq. phos. ammoniæ...
Aquæ...... ...............aa oz. ij M.

Sig.—Tablespoonful thrice daily.

The solution of phosphate of ammonia is of the strength of 60 grains to the ounce of water.

CHOREA.

Dr. Maris depends upon cimicifuga for treating this convulsive disorder. He has cured many cases with this drug. Arsenic is sometimes prescribed, but it has not met his expectations; he thinks it may be due to his not pushing the arsenical treatment sufficiently.

Iron is given in anæmic cases, and every source of irritation removed. The doctor is now treating a case of chorea in a young lady twenty-one years old, whose left foot is the seat of the affection. The movement is of a stamping character, and has worn out the shoe on this foot. The treatment is by cimicifuga, but she has not been using it long enough to note the effect.

Some of the house prescriptions used in this institution are as follows:

I. MISTURA INTERMITTI.

R. Cinchoniæ sulphatis.....gr. xvj
Acid. sulphuricum......gtt. ij
Tinct. ferri chlor.........f. dr. j
Syrupi...... ........,......
Aquæ font................f. oz. j M.

II. MISTURA FUSCA.

R. Ext. glycyrrhizæ ....... gr. xv
Pul. acaciæ,
Sach. albi............ ...aa gr. xv
Tinct. opii camph.........f. dr. j
Vinum antimonii.........f. dr. ss
Spts. ætheris nit.........gtt. xv
Ammoniæ murias.......gr. xv
Aquæ.......................f. oz. j M.

III. MISTURA TUTTII.

R. Ol. morrhuæ.............f. ox. ij

Tinct. ferri et quassiæ..f. oz. ss M.

IV. MIST. POTASSII CHLORATIS ET FERRI.

R. Potassi chloratis.........dr. ss
Tinct. ferri chlor.........f. dr. j.
Aquæ, q. s. ad......f. dr. j. M.

V. PILULA PODOPHYLLI COMP.

R. Podophilii.....................gr. ss

Ext colocynth comp......gr. ijss

Ol. carui,......................gtt. ¼ M.

Et ft. pil. No. 1.

VI. PIL. OPII COMPOSITA.

R. Pulv. opii.........................gr. ss
Mass. hydrarg..... ........,gr. j M.
Et. ft. pil. No. 1.

VII. PIL. CERII OXALAS.

R. Cerii oxalas.......................gr. j
Ex. gent., ... .................q. s. M.
Et ft. pil. No. 1.

VIII. PULVIS COLUMBÆ COMPOSITUS.

R. Columbæ.......................dr. iv
Mag. carb....................dr. iij
Zingiberis.....................dr. j
Ol. carui.......................gtt. iv M.
Sig.—A half drachm night and morning.

IX. SYRUPUS CERASI COMPOSITUS.

R. Morphiæ acet.. .............gr. j
Syr. scillæ............ .... ..f. oz. j
Syr. pruni virg.............oz. ij M.
Sig.—One drachm three times a day.

X. SYRUPUS ASSAFŒTIDÆ.

R. Tinc. assafœtidæ......f. oz. ss

Syrupi.......... .........oz. iss M.

XI. SYRUPUS PECTORALIS.

R. Syr. pruni. virg...........,f. oz. j

Tinc. Benzoni comp, ...

Tinc. hyoscyami.....aa f. oz ss M.

Sig.—A teaspoonful three times a day.

XII. SYRUPUS ASTHMATICUS.

R. Syr. scillæ.............f. oz. j
Tinc. lobeliæ.........
Liq. morph. sul......aa f. oz. ss M.
Sig.—A teaspoonful three times a day.
—*Med. and Surg. Rep.*

